# Strategies and behaviors used by mothers in interactions with their young children during a mealtime in peri-urban areas of Huánuco, Peru

**DOI:** 10.1016/j.dialog.2022.100034

**Published:** 2022-08-11

**Authors:** Sissy Espinoza-Bernardo, Rosario Bartolini, Hilary Creed-Kanashiro, Doris Delgado-Pérez, Emma Haycraft

**Affiliations:** a Departamento de Nutrición, Facultad de Medicina, Universidad Nacional Mayor de San Marcos, Peru; b Instituto de Investigación Nutricional, Peru; c Instituto de Investigación de Bioquímica y Nutrición, Departamento de Nutrición, Facultad de Medicina, Universidad Nacional Mayor de San Marcos, Peru; d School of Sport, Exercise and Health Sciences, Loughborough University, UK

**Keywords:** Responsive feeding, Mealtime interactions, Parent-child interactions, Encouragement to eat, Pressure, Distractions

## Abstract

This study aimed to gain an in-depth understanding about different strategies used by mothers to feed their 6-23-month-old children, as well as to learn about mothers’ behaviors in response to situations of food refusal by her child, in order to generate evidence that contributes to the information gap on responsive feeding in Peru. The study was conducted in the city of Huánuco, a peri-urban area of Peru, with mothers of children in the complementary feeding stage participating. An in-depth 5-hour home observation was conducted in eight mother-child dyads. Both the mothers’ and children’s mealtime and food-related behaviors were coded and an inductive thematic analysis was applied. The primary objective of many of the strategies used by the mothers was to get their child to eat a little more. Six strategies were identified: pressure, encouragement, facilitating intake, acceptance, negotiation and reasoning. Certain differences were found in the strategies employed by the mother according to the age of her child, with mothers of younger children using more encouragement and mothers of older children using more pressure for their child to eat. The mothers’ behavior in response to the child's refusal of food was both responsive and non-responsive (controlling), depending on the reason for the refusal. The findings are of great value for understanding about the feeding interactions of mother-child dyads in Peru and they start to address the information gap and can support the development of nutritional intervention strategies for use with children.

## 1 Introduction

Responsive feeding involves responding to infants’ and children’s hunger and satiety cues, feeding with patience, helping children to eat autonomously, and encouraging them to eat without forcing them [1,2]. Responsive feeding allows for an appropriate interaction between the caregiver and the child which can result in a positive feeding experience, adequate food intake and enhanced developmental opportunities [3]. It is currently recognized that malnutrition in its different forms can be reduced when applying optimal interactions between caregiver and child during feeding episodes, with responsiveness, supporting the child's development of self-feeding, and providing a supervised and distraction-free feeding environment being core components of this [4,5]. Responsive feeding strategies have been associated with generally positive responses from the children, whereas food parenting styles which are overly controlling can have a negative impact on the child's development and eating behaviors by interfering with their autonomy and disrupting their eating habits and wellbeing [6].

Research suggests that non-responsive maternal feeding behaviors use high levels of force or control over the young child and provide few opportunities for self-feeding [3,5]. Likewise, controlling behaviors such as pressure, force and negative verbal expressions have been associated with children’s refusal of food, less consumption of food, and increased negative perceptions towards food [[7], [8], [9]] or with children engaging in passive eating behaviors [10]. On the other hand, a receptive interaction accompanied by positive verbal expressions is associated with higher rates of children’s acceptance and food intake [3,11,12]. It is therefore important to understand the strategies used when feeding, and also the mothers’ behaviors in difficult situations when feeding her child, since they are related to children’s food intake and could affect the growth, wellbeing and development of the child.

While there is an abundance of literature which has explored feeding behaviors in countries such as the USA, UK and Australia, there is less evidence into the responsive feeding practices of parents across cultures [7,13,14]. In Peru, for example, there is limited evidence exploring the use of responsive feeding practices or understanding the experiences and behaviors of mothers when they are faced with various child behaviors that present during feeding (such as food refusal, tantrums, rapid eating). A study carried out in a rural area of  Lima showed greater use of responsive feeding behaviors in multiparous mothers, as opposed to primiparous mothers who typically acted in a more authoritarian manner when their child rejected food [15]. Another study, carried out in the city of Huánuco, reported a low level of responsive feeding practices, indicating that these mothers were mostly unaware and did not practice responsive feeding [16]. Together, these sparse findings suggest that more research is needed to understand the feeding behaviors of mothers and other caregivers in Peru, particularly in peri-urban areas where there seems to be a lack of responsive feeding, in order to inform nutrition promotion programs and interventions within Peru.

The present research aims to contribute to this information gap by describing and understanding in depth the feeding strategies and behaviors of mothers during feeding time and in situations of food rejection by their young children, in families living in a peri-urban area of Huánuco. Peru. These findings will be useful in informing and better targeting nutritional intervention strategies aimed at promoting healthy child nutrition, development and wellbeing [[17], [18], [19]].

## 2 Methodology

A descriptive qualitative study was conducted, the purpose of which was to explore and describe the behaviors of mothers relating to responsive feeding and to identify the strategies used by the mother when feeding her child. The population consisted of mothers of children between 6 and 23 months of age, living in peri-urban areas of the city of Huánuco: Loma Blanca, Vista Alegre, Cruz Verde and Las Lomas, pertaining to the jurisdiction of the Aparicio Pomares Health Center in the district of Huánuco, Peru.

### 2.1 Participants

Eight mother-child dyads were included in the study. The participants were selected by purposeful sampling according to the following criteria: a mother in charge of feeding a child aged 6 to 23 months, and who had previously participated in the Instituto de Investigación Nutricional (IIN)'s PERU SANO project, had lived for at least one year in the study area, and voluntarily accepted to participate in an observation in their home.

### 2.2 Ethical procedures

The study was approved by the CIEI (research ethics committee) of the Instituto de Investigación Nutricional, Peru, with the number: 388-2019/CIEI-IIN. All participants were informed of the observation procedure, that all information provided would remain confidential, and the benefits and risks of the study, prior to signing an informed consent form.

### 2.3 Procedure

The data were collected during February and March 2020. Observations in the natural environment were conducted in the child's home, with an average duration of five hours which included mealtime preparations and the moment of eating (mealtimes). In most cases, the time observed was almost five hours in the morning (from 8:30 am to 1:30 pm), which included breakfast, mid-morning snack and lunch (which is typically the main meal of the day in Peru). The day and time of the observation was agreed upon with the mother in a previous visit where the objective and procedure to be carried out was arranged. On the day of the observation, the mother signed the consent form for the observation and for taking photos to record events which occurred. The mother was asked to continue with her normal daily activities and the researcher explained that they would not interrupt nor interfere with the mother’s activities. The researcher was positioned to observe and take notes of all the situations relating to the feeding processes and she moved discreetly during the observation, if required, to capture certain behaviors. An observation guide was used to direct the researcher's focus of attention to the relevant events and to record what, how, when and who participated in feeding the child. The physical characteristics of the environment, actions and situations that occurred at the time of feeding the child were recorded.

The information obtained from the observation was complemented by photographs of the entire process which made it possible to subsequently evoke memories of the observation and capture critical moments. The data were collected by two researchers, a nutritionist and an anthropologist, both of whom participated in the creation of the observation guide and developed the study design. The observation guide comprised two sections; the first of which was for recording observations of the context, describing the home environment and the environment for feeding the child. The second referred to the actions that the mother performed to feed the child and all the actions that the child performed before, during and after eating. The same observational procedure was followed for each family. Each observation was recorded in the field log, then transcribed using a word processor. Each transcribed file was collated alongside the photos of each mother and child observed and organized into folders for analysis. On completion of all the observations, the transcripts were analyzed by the first author.

### 2.4 Analysis of data

The data analysis began with a review and reading of the field log. In order to organize and systematize the information, matrices in Excel datasheets were used: one with all the information collated by themes, the second with mothers' behaviors and a third with the child's behaviors during the child feeding event (meal/snack time). The information was organized and systematized according to the principles of thematic analysis [20]. This information was presented diagrammatically to facilitate interpretation of the findings. Tables were also constructed, in which illustrative quotations for each category/theme were placed. In addition, the researchers reviewed and analyzed each matrix to reach a consensus on the strategies used by the mothers.

## 3 Results

### 3.1 Characteristics of the mother-child dyads

The average age of the eight mothers who participated in this study was 32 ± 4.89 years, of which half of them had only one child and the other half had more than one child. The children were aged from 6 to 23 months with an average age of 14 ± 4.76 months; three were girls and five were boys.

### 3.2 Strategies used by mothers during the feeding events (meal or snack times) of children aged 6 to 23 months

The mothers were observed to use various strategies to encourage their child to eat and finish the food served or at least, to eat a little more when the child did not finish. These strategies were grouped and categorized as either pressure, encouragement, facilitating intake, agreement to stop eating (acceptance), negotiation, and reasoning. Table 1 shows examples of the verbal expressions and physical behaviors of the mother corresponding to each of the strategies used while feeding her child.

**Table 1:** Verbal and non-verbal interactions (expressions and behaviors) observed during mealtime with a child aged 6 to 23 months.

Categorized strategy or behavior	Characteristics	Example of verbal expressions	Example of behaviors
Pressure	Indication, Insistence.	*"Open your mouth, open your mouth" (Ma13m); "Sit down, finish your plate" (Ma21m); “One more, but you haven't eaten anything, you have eaten a little”(Ma17m).*	*The mother, seeing that her child has eaten very little, chases him with cup in hand until he finishes eating (Ma13m).*
	Warnings, Control	*"Eat my son,orthe cat eats your food ... I don't know" (Ma20m); “Junior, finish your meal… I'm going to be upset" (Ma21m).*	*With just the look of the mother, the child sits in his chair, takes the spoon and begins to eat some more (Ma21m).*
	Threat, force	*"If you don't finish, I won't take you to the street, you stay" (Ma21m); "Ah..you ate little, I'm not going to put you down" (Ma12m).*	*The mother puts the spoon in the child's mouth, although the child does not pass the food, by pressure the child accepts until he can no longer take it and vomits (Ma13m).*
Encouragement	Demonstrations of love	*The mother gives her a spoonful, "now my love"and the girl receives(Ma17m); "Come my love, come eat, eat, grab Jean" (Ma21m).*	*The mother with* a *very smiling face feeds him andthe baby looks at her and smiles at her (Ma20m).*
	Priority to a food	*"Very good, eat everything, all your little fish" (Ma21m), "leave me the soup, finish your second" (Ma21m).*	*Richy's mother offers him the second course as the main meal (Ma20m).*
	Food attributes	*"how is it? rich? rich?” (Ma17m);"Eat son ... the soup is delicious" (Ma21m).*	*Kiran's sister makes the gesture of liking and wanting to eat the spoon of her food (Ma10m).*
	Praise	*"Emmy, you are close to finishing, bravo" (Ma11m); "very good Kev, you finished your second" (Ma21m).*	*The mother smiles at the child in satisfaction and congratulatesthat he finished his meal (Ma20m).*
	Actions to encourage eating	*The mother says, "eat eat, the plane landed, the plane landed on Kiran's belly" (Ma10m).*	*Mothers carry out environmental actions: clap, sing, play, listen to music, watch the animals feed.*
	Actions to distract	*“Emmy, what is this? ... elephant and what is this?” (Ma11m);"Every time he shows the puzzle shape to him, he gives him a spoonful of food”(Ma08m).*	*Use of distractors: grabbing food, objects and/or toys, children's videos on cell phones or television*
Facilitating intake	Offer liquid with food	*"More water, more water for it to pass" (Ma13m).*	*During the meal, the mother offers her a glass with granadilla soda so that she can pass her meal (Ma08m).*
	Improvements to flavor and texture	*"Ah ... sure you want more honey ... you are known, Emmy" (Ma11m); "I needed to add the little oil to improve the flavor and make it smoother" (Ma08m).*	*To improve the taste and consistency of the porridge, the mother adds olive oil (Ma08m); a caregiver adds honey to the girl's oatmeal because she likes it that way (Ma11m).*
	Offers appropriate food size	*"Take your little fish, and you finish it all" (Ma20m).*	*Ray's mother offers the boy a piece of apple cut in such a way that he can pick and eat on his own (Ma13m).*
Agreement to not eat any more (acceptance)	Recognizing cues	*"She will not eat any more" (Ma12m);"I don't insist on eating,* so *he doesn't vomit" (Ma08m).*	*When the mother sees that her son holds food, does not pass it, throws it and wants to get up from the chair, she recognizes signs of not wanting to eat and lets him go (Ma13m).*
Negotiation	Agreement between both	*"You can leave the soup, but you finish me second" (Ma21m); "Leave the rice, but you finish all the fish for me" (Ma20m).*	*The mother insists that he eat the egg, makes him taste it with his hand and then the child eats the whole egg, but leaves the oats (Ma21m).*
Reasoning	Food and health relationship	*“Eat eat, so that you grow big, do you want to be big or small?" (Ma10m).*	*Mothers recognize that children must first eat and then breastfeed, because they fill up with breast milk or fall asleep and no longer want to eat. (Ma11m).*

The pressure strategies observed were applied by the mother with the intention of getting the child to eat. Pressure refers to the characteristics of insistence or threat. In the observations, insistence was exhibited as firmness and constancy from the mother for the child to finish the meal/snack. Warnings or threats were also made by the mother for the child to finish their meal as well as instances of the mother forcing the child to finish, regardless of whether the child appeared to be accepting the food or not. Examples of pressure strategies can be seen in Table 1.

Encouragement strategies included behaviors used with the intention that the child continues eating but these were not forceful (see Table 1). The various ways that the mother encouraged her child to eat included: prioritizing that the child eat a particular food, verbal expressions about the attributes of a food (“rich”, “tasty”), praising the child at the end of the meal, using actions or games to encourage eating, and distracting the child. Distraction strategies were used to attract the child's attention or to entertain him/her and thus take advantage of the child’s distraction to feed him/her. Distractors used included mothers picking up objects and/or toys (this strategy was very commonly used) and also feeding the child while the child is busy with an object/container and is exploring the object with their hands. Mothers also used the television or cell phones to distract the child and feed whilst the child was watching the screen.

Strategies of modifying the food were also used by the mother to facilitate the child's intake, such as providing liquids to the child while eating to help the child swallow solid foods and preparing foods in a way preferred by the child to improve the taste and/or texture. For example, one mother used honey to improve the taste of oatmeal and her daughter was observed to like it. Another mother used olive oil to improve the taste and texture of the lentil stew. Some mothers made it easier for the child to pick up the food by cutting the food into an appropriate size for the child to grasp and put it into their mouth.

Agreement was evidenced when the mother recognizes the child's signs of satiety or perceives that her child is not going to eat anymore, for example, by withdrawing or wanting to get down from his/her chair, grabbing his/her tummy or removing his/her plate, by turning his/her face away or showing a sensation of regurgitation or vomiting. When faced with this, mothers often no longer insisted or pressured the child to eat but accepted that the meal was finished. This behavior was observed when the child was full, because the mother recognizes that the child has eaten at different times or has had enough. In that case mothers either kept the food for later or removed the food to discard.

Negotiation was observed when mother and child came to an agreement about the food to be eaten. This was often based on an alternative proposal, or a compromise, with the intention of still fulfilling the objective of the child consuming some (more) food. For example: *"you can leave the soup, but you finish the fish"*.

The reasoning strategy captures when the mother explains to the child the relationship between food and health or growth. For example, *"Eat, eat, so that you grow big, do you want to be big or small?”*. However, this strategy was only used very rarely by the mothers in the study.

A further aim of this research was to understand if there were any differences observed in the feeding strategies used according to the age of the child. For this analysis, the dyads were split into two age groups: group one comprised mothers with children aged 6 to 12 months (n = 4) and group two comprised mothers with children aged 13 to 23 months (n = 4).

**Fig. 1:**
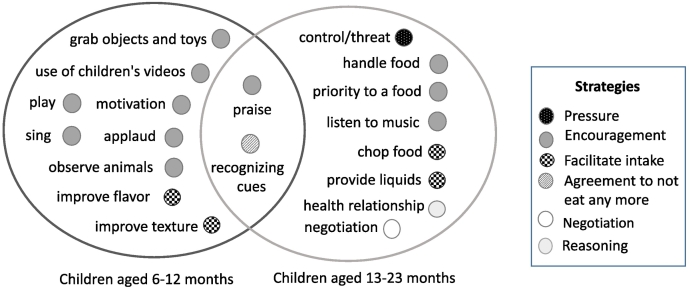
The frequently observed strategies and behaviors that mothers use while feeding their children, differentiated by the age of the child (6–12 months; 13–23 months).

Fig. 1 shows the frequent strategies and behaviors that mothers used when feeding their child. In this figure the different and common actions used by the mothers according to their child’s age are identified.

For the younger children aged 6 to 12 months, mothers tended to use more encouragement strategies, mainly those related to environmental actions to encourage eating such as: singing, playing, clapping and, to a lesser extent, distraction strategies such as picking up objects and/or toys or children watching videos on cell phones or the television.

The difference in the strategies used by the mothers was that mothers of the older children (aged 13 to 23 months) were observed to use the pressure strategy, which included them using more warnings and threatening actions with their children. Another difference was that mothers of older children were more likely to give their child the opportunity to handle food with the intention that they feed themselves. In this group of mothers, other strategies were observed that were not present with the younger ones, such as the use of reasoning that aimed to stimulate in the child an interest in food and explain its health benefits. Reasoning with the older children also included encouragement for the child to do something active, with the intention that the child gets tired, rests and then has a greater appetite for food.

The common strategies observed in both groups of children were encouragement (praise) and mothers’ agreement that the child will not eat anymore (recognizing cues) See Fig. 1.

### 3.3 Mothers’ behavior in response to food refusal situations that arise when feeding a child aged 6 to 23 months

The final part of the analysis aimed to understand the mothers’ behavior when faced with the rejection of food by her child. There were several diverse reactions observed that appeared to be related to the child’s age, distraction and satiety. Both responsive and non-responsive feeding behaviors were observed in mothers in response to some of the various reasons why the child refuses to eat, as shown in Table 2.

**Table 2:** Mothers' behavior when the child refuses food when distracted, satiated or does not want to continue eating.

Situations	Examples of the mother's observed behavior	
	Responsive feeding	Non-responsive feeding
Child gets distracted	• Supported continuous feeding and regained the child’s attention.	
	• Encouraged the child to continue eating using verbal and non-verbal expressions of affection and attributes of the food. • For younger children, stimulation strategies to eat such as singing, clapping were applied.	• Discourages or did not allow the child to self-feed. • In older children, use of reprimands or raised voice (but without offensive words, without physical aggression).
Child is full or does not want to continue eating	• The mother paused feeding and persisted at another time.	• They used distractors with the intention that he continues to eat such as videos on cell phones or television and that he picks up objects. • They used controlling actions such as: following the child with food, force-feeding him/her, using verbal and non-verbal expressions of pressure or threat.
	• They facilitated the child’s intake by improving the taste and size of the food. • They used encouragement strategies, priority given to certain foods, verbal expressions to highlight attributes of a food. • They used congratulatory strategies when the child was making progress and when they finished their meal; or negotiation and reasoning in older children.	

In situations where children were distracted and stopped eating, it was observed that mothers used a variety of behaviors and often responded differently depending on the age of the child. For younger children, mothers tended to use encouragement and distraction strategies while the mother was feeding the child. In some cases, they limited self-feeding and took over feeding the child themselves. While older children (over 12 months) were more independent, the mother tried to return the child's attention to the food with verbal or nonverbal expressions of insistence or threat. These mothers often used more non-responsive behaviors; in some cases, they spoke loudly, but without offensive words or physical aggression.

When the child showed signs of satiety or did not want to continue eating, mothers were observed to show both responsive and non-responsive behaviors. For example, they sometimes understood and accepted that the child had already eaten enough, did not pressure the child, and let the child leave the meal/snack. However, it was also observed in some mothers that when the child no longer wanted to continue eating and the child showed signs of refusal, that the mother insisted that the child eat, used verbal expressions of pressure, or forced the child to eat to the point that the child sometimes regurgitated (or vomited) the food. In these more extreme cases, it was observed that the mother ultimately resigned herself and let it go.

## 4 Discussion

This research aimed to understand mothers’ use of feeding strategies, and mothers’ behaviors in response to food refusal situations that occur whilst feeding a child, using a sample of mothers and children aged between 6 and 23 months living in peri-urban areas of Huánuco, Peru. The findings revealed the use of diverse strategies by the mothers during their children's meals which were often driven by the intention of getting children to finish their food. These strategies included the use of both responsive and non-responsive behaviors when the mother faced food refusal situations during the process of feeding her child.

### 4.1 Strategies used by mothers during the feeding events (meal or snack times) of children from 6 to 23 months

The feeding strategies used by the mother when feeding her child were primarily aimed at getting the child to eat and to eat a little more. This aligns with the research of Orrell-Valente et al. [21] who found that in their USA sample, regardless of the socioeconomic status of the families or the sex of the child, the main goal of most parents is to get children to eat more during mealtimes. In the present study, the mothers used different strategies such as encouragement, facilitating food intake, pressure, agreement not to eat any more, negotiation and reasoning. Notably, this study observed the use of predominantly responsive feeding behaviors. Encouragement, a responsive practice, was the most widely used strategy in this group of mothers and it took the form of both actions and verbal expressions which aimed to encourage the child to eat. Several studies reaffirm the use of this strategy to improve the child's intake [14,22] and its use is related to supporting children’s healthy development around food, eating and mealtimes.

Mothers implemented numerous strategies to facilitate their children’s intake of food in this study, and these tactics appeared to contribute to a greater acceptance of food. This is likely because these practices involved acknowledging the child's food preferences, improving and varying the taste and appearance of some foods, and cutting up foods to enable the child to pick them up and self-feed. These findings expand on past evidence of facilitation from a study by Lebron et al. [23] with Hispanic families living in the USA who found that, when introducing new foods or foods that the child did not like, caregivers often chose to hide disliked/new foods or improve the taste with other foods so that the child would accept them. They also extend the findings of another study conducted in a rural area in Peru which identified sensitive caring behaviors in mothers when caring for her infant [24] by revealing the use of responsive feeding behaviors in Peruvian mothers.

The strategies of negotiation and reasoning were often used by mothers of children older than 12 months. Negotiation was typically observed as an agreement between mother and child in an attempt to get the child to consume a food or meal and is likely to be more effective with older children, whose development has progressed meaning that negotiation can be effective. Such a practice is responsive, accommodating the child’s wishes, and extends previous evidence into the use of bribery, a less responsive practice where a child is incentivized with something to finish the food/meal [23,25], which has been shown to contribute to the subsequent development of overeating and overweight.

The strategy of acceptance occurred mainly in mothers of children older than 12 months, when the mother recognized the child’s signs of satiety and accepted that the child will not eat more. It is likely that mothers are better able to interpret the satiety cues of older children and that these children are better able to communicate them, either verbally or with gestures. Studies have shown that the response of children to hunger and satiety signals can be affected by external signals, such as controlling strategies of mothers [26], which can lead to overeating in response to the demands of caregivers [21], and to the inability to control food intake [4]; both of which can contribute to overweight or obesity. What is evident in the current study is that mothers were able to feed responsively, often responding appropriately to their child’s satiety cues.

Some mothers in this study also used pressure, a non-responsive (or controlling) practice. Children's behaviors can influence the strategies used by the mother, mainly in the way she conducts herself [21]. The current study found that when the mother exerts pressure, such as insistence and threats or uses some forceful action on the child, the child often responded by eating one or two more bites but still did not finish the food. This was often accompanied by the child expelling the food from his/her mouth, vomiting the food or refusing to eat it, and the child was often visibly distressed during this. Likewise, when mothers used a visual or physical signal, such as gesturing or a facial expression, as an indication or a reminder for the child to eat, children typically ate a little more but did not finish their food. These findings suggest that the use of pressure was not generally associated with children finishing their meal and that it often had adverse, unintended consequences.

### 4.2 Feeding strategies according to the age of the child

This research found certain differences in the feeding strategies used by the mother according to the age of her child. This supports the idea that caregivers should adapt and adjust their feeding behaviors according to the age and developmental stage of their children.

Mothers of the younger children aged 6 to 12 months were able to comply with some dimensions of responsive feeding [5], such as encouraging the child appropriately when they had not eaten enough and talking to the child (without ordering or pressuring him or her) during meals. Encouragement and distraction were frequently used with this age group. There were fewer instances in this group of mothers allowing the child to self-feed, opting instead to feed their child and often not allowing the child to take the food. This corroborates the findings of Bautista [16], where the majority of the mothers in a Huánuco community never allowed their young child to use their hands to eat or use a spoon. Not allowing children to feed themselves, especially if it is delayed for more than 24 months [3,5], can limit their development. Horst et al. [6] noted that such overprotection used with young children can interfere with their development of autonomy.

A marked difference in the strategies of mothers of children older than 12 months was to give their children more opportunities to feed themselves. However, this was also accompanied by a greater use of pressure strategies characterized by control and force, expressed both in behaviors and in verbal and nonverbal expressions. Importantly, Aboud et al. [17] indicate that the use of pressure strategies may lead to short-term compliance, but does not help children develop a healthy appetite, recognize hunger and satiety cues, or eat at an appropriate rate. In this group of mothers, it was also observed that there was evidence of some engaged feeding of the child, but it was often not responsive; children were sometimes left to eat alone and there was little physical and verbal interaction, and very little stimulation of the child during feeding time. This is consistent with the findings by Bentley et al. [27] in which caregivers offered little physical assistance with eating, as they felt that the children "know" how much to eat, so they let them be more independent and rarely verbally encouraged them to eat. Quite the opposite was found in a study in Vietnam [3], where feeding is still supported 70% of the time up to 18 months and there is a greater willingness of the mother to sit with her child and verbalize during feeding. Similar results have been found in other settings [12,13]. The findings in this Peruvian sample therefore show a mixture of engaged, sometimes controlling practices, alongside more permissive behaviors; both of which can be linked to less favorable eating outcomes in children and are therefore important to target in health and wellbeing intervention programs for families.

One common strategy observed in both age groups of children was positive verbal expressions (congratulations, praise) that are seen as encouragement strategies, based on positive reinforcement, as established by Dearden et al. [22] and Engle et al. [28] who both found that verbal encouragement from caregivers can increase children's acceptance of food. Mothers of younger and older children facilitated their child’s intake of food, but with different means; in the case of younger children, mothers tended to improve the taste and texture of the foods, and in the case of older children they facilitated ingestion by providing fluids to pass through the food and by chopping the food so that the child could pick it up. Such behaviors are age-appropriate and will likely support children’s healthy developing relationship with food.

### 4.3 Mothers’ behavior when their 6- to 23-month-old child refuses food because of distraction, satiety, or not wanting to continue eating

The mothers’ behavior was often observed to be a reaction to the refusal of food by their 6-23-month-old child. Mothers reacted differently according to the perceived reason for the food refusal and the age of the child; where mothers felt the child was distracted, some mothers motivated and supported them in feeding, particularly with the younger children, while others were more intrusive and did not allow the older children to self-feed. With the older children, the mother was more likely to pressure them to finish their meal, calling their attention to the food or pressuring them in different ways. However, when the child’s cues suggested that he/she was satiated, the mother's reaction was often different. Some mothers accepted what their child had consumed, while others insistently pressured the child to eat more, but this often had the opposite effect and perpetuated the child’s food refusal. Where children were refusing food due to discomfort or feeling unwell, their mothers were observed to show greater interaction and concern for the child to finish his food, even if it was little by little. Together, these observations suggest that not only are mothers’ reactions different based on the age of the child but also based on the reason for the food refusal, with evidence of some sensitive, responsive feeding occurring in many instances but a tendency for pressure to be used, particularly in response to food refusal by older children.

### 4.4 Strengths and limitations

Strengths of this research include the in-depth, detailed observation of families’ eating occasions across a number of hours, the coding of both maternal and child behaviors, and consideration of child age as a factor impacting the feeding interactions. Further strengths lie in the detailed description of the various strategies used at the time of feeding a child, and understanding of the interaction between mother and child (i.e., understanding responses to the other’s behavior). All of this addresses the existing information gap regarding the level of responsive feeding in Peru and contributes to better consideration of the need for food/nutrition interventions in this group of mothers or caregivers responsible for feeding the child. A limitation of the research includes the sample size which was relatively small, although it is acceptable for qualitative studies [29] and both the saturation of the data and the richness of the information were considered, and also the focus just on one geographic area of Peru. Further work is required to build on these findings with more diverse groups.

## 5 Conclusion

In conclusion, the feeding strategies used by mothers with children were diverse, depended on the age of the children and on the feeding situation. There was evidence of both responsive, attentive caregiving as well as less sensitive, more controlling feeding at times. The mothers’ behaviors in the face of food refusal or the child's lack of appetite were often not very responsive, while the mothers’ behaviors with the younger children, under one year of age, were more permissive and used greater dimensions of responsive feeding. Notably, mothers adjusted their behaviors based on child factors, such as satiety cues or the child not wanting to continue eating. This study has made important advances in identifying the diversity of strategies used by mothers or caregivers when feeding children in Peru during the period of complementary feeding; a critical age when responsive feeding can contribute to both optimum nutrition and development. Interventions are recommended to help improve the strategies and behaviors used by mothers in difficult feeding situations. At the same time, encouragement of responsive feeding and the division of responsibilities for healthy feeding is recommended.

## Funding

UK-Peru Newton Fund with the UK Medical Research Council (MR/S024921/1) and CONCYTEC/FONDECYT Perú (032-2019). The funders had no involvement in the study design, conduct, analysis or reporting.

## Declaration of Competing Interest

The authors have no conflict of interest to declare.
